# Overexpression of *CsMIXTA*, a Transcription Factor from *Cannabis sativa*, Increases Glandular Trichome Density in Tobacco Leaves

**DOI:** 10.3390/plants11111519

**Published:** 2022-06-06

**Authors:** Samuel R. Haiden, Peter V. Apicella, Yi Ma, Gerald A. Berkowitz

**Affiliations:** Department of Plant Science and Landscape Architecture, Agricultural Biotechnology Laboratory, University of Connecticut, 1390 Storrs Rd. Unit 4163, Storrs, CT 06269-4163, USA; samuel.haiden@uconn.edu (S.R.H.); apicellapv@gmail.com (P.V.A.)

**Keywords:** *Cannabis* *sativa*, glandular trichome morphogenesis, transcription factor, cannabinoid biosynthesis, female flower development

## Abstract

Cannabinoids are synthesized in glandular stalked trichomes on the female flowers of *Cannabis sativa* (cannabis). The regulation of glandular trichome development has not been characterized in cannabis. We recently identified an R2R3-MYB transcription factor, *CsMIXTA*, which could be involved in trichome morphogenesis in cannabis. Some homologous genes of *CsMIXTA* are known to function in glandular trichome initiation in other plant species. *CsMIXTA* is highly expressed in flower tissue compared to vegetative tissues. Interestingly, *CsMIXTA* is also highly expressed in trichomes isolated from female flower tissue. In addition, *CsMIXTA* is upregulated during the peak stages of female flower maturation in correlation with some cannabinoid biosynthetic genes. Transient expression in *Nicotiana* *benthamiana* showed that *CsMIXTA* is localized in the nucleus. Furthermore, yeast transcriptional activation assay demonstrated that *CsMIXTA* has transactivation activity. Overexpression of *CsMIXTA* in *Nicotiana tabacum* resulted in higher trichome density, larger trichome size, and more branching on stalked glandular trichomes. The results indicate that *CsMIXTA* not only promotes glandular trichome initiation in epidermal cells, but also regulates trichome development in tobacco leaves. In this report, we characterized the novel function of the first cannabis transcription factor that may be critical for glandular trichome morphogenesis.

## 1. Introduction

Glandular trichomes are epidermal outgrowths characterized by the presence of a secretory cavity or “gland”, which houses secretory gland cells capable of synthesizing secondary metabolites and exporting them into a secretory reservoir [1,2]. Glandular stalked trichomes (GSTs) on cannabis female flowers emerge from an epidermal cell and develop multicellular stalks which are usually less than 500 µm long [3,4,5,6]. In cannabis, glandular trichomes contain a rosette of secretory cells that synthesize and export biosynthetic enzymes into the secretory reservoir [7,8]. The direct precursor of cannabinoids, cannabigerol (CBG), is synthesized in the plastid of the secretory gland cell, and then exported (by an unknown mechanism) into the secretory reservoir, where it is converted to tetrahydrocannabinolic acid (THCA), cannabidiolic acid (CBDA), or cannabichromenic acid (CBCA) by their respective synthases [7].

Current models indicate that the regulation of secondary metabolite production is associated with the regulation of flowering and trichome development [9,10]. After an epidermal cell receives a signal to initiate trichome morphogenesis, it develops into a sessile glandular trichome [1]. In concert with the maturation of the flower, sessile trichomes will develop into the bulbous phase and then eventually reach the capitate-stalked glandular (CSG) phase [11]. This is the mature phase at which cannabinoids are predominantly produced [10].

A complex of transcription factors (TFs) in the R2R3-MYB and HD-ZIP IV families has been shown to regulate the development of glandular trichomes in other plant species [11,12,13]. This complex includes MIXTA-like proteins, which interact with other HD-ZIP IV TFs to coordinate trichome development in concert with flower development [13]. Understanding the genetic regulation of the development of a glandular trichome from sessile to stalked is of particular importance for cannabis biology because it has been shown that cannabinoids are not produced in any considerable amount until the trichome reaches the CSG phase [10].

In cannabis, the genetic regulation of glandular trichome initiation and development is still unknown. In this study, we identified a novel R2R3-MYB TF in cannabis, *CsMIXTA*, which could positively regulate cannabis CSG development. We showed that *CsMIXTA* was highly expressed in flowers; *CsMIXTA* also showed higher expression in trichome cells isolated from female flower tissue. Ectopic expression of *CsMIXTA* in tobacco not only significantly increased glandular trichome size and density but also promoted trichome branching on tobacco leaves. Our study suggests that *CsMIXTA* is a TF that could potentially control glandular trichome initiation and development in female cannabis flowers.

## 2. Results

### 2.1. CsMIXTA Is Upregulated during Female Flower Development

**Figure 1:**
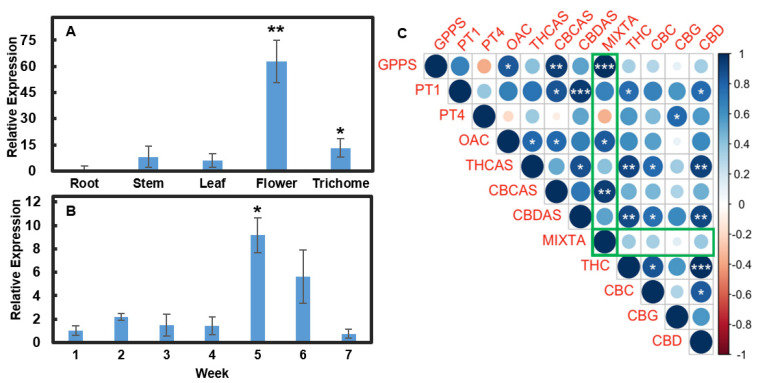
Expression analysis of *CsMIXTA*. (**A**) Relative expression of *CsMIXTA* in 5 different tissue types harvested from Cherry Wine. (**B**) Relative Expression of *CsMIXTA* over 7 weeks of flowering. Results ((**A**,**B**)) are shown as means ± SE (*n* = 4). Means separation between expression in various tissues compared to the level in roots (**A**) and at various times during flower development as compared to expression at week one (**B**) was evaluated using Student’s *t*-test; * indicates *p* < 0.05, and ** indicates *p* < 0.01. (**C**) Integrated expression analyses of *CsMIXTA* and genes encoding cannabinoid biosynthetic enzymes along with cannabinoid levels monitored over the 7-week flowering period were evaluated using Pearson’s correlation coefficient analyses. Green boxes highlight *CsMIXTA*. Blue indicates a positive correlation, while red indicates a negative correlation. Size and shade of circles represent strength of correlation. Figures were generated using the R corrplot package (https://github.com/taiyun/corrplot). (*, *p* < 0.05; **, *p* < 0.01; ***, *p* < 0.001.) Percentage data was arcsine transformed prior to statistical analysis.

MIXTA has been reported to be involved in glandular trichome formation in various plant species, such as snapdragon and artemisia [12,14]. We searched the NCBI database using the known MIXTA sequences from snapdragon and artemisia to identify a homolog in *Cannabis sativa*. We named the cannabis gene CsMIXTA. We first determined *CsMIXTA* expression in different tissues and during female flower development in a hemp variety, Cherry Wine (CW). *CsMIXTA* is highly expressed in flower tissues compared to the vegetative tissues (Figure 1A). *CsMIXTA* is also expressed in stem and leaf tissues, where trichomes are also present. Interestingly, *CsMIXTA* was detected in isolated trichomes, suggesting that *CsMIXTA* may have distinct functions in processes other than trichome initiation. We further examined *CsMIXTA* expression during a period of 7-week flower development. The results showed that *CsMIXTA* was significantly upregulated in week 5 and reduced thereafter (Figure 1B). *CsMIXTA* showed a similar expression pattern to that of the enzymes in the cannabinoid biosynthetic pathway [15]. We used a Pearson’s correlation matrix test to evaluate the relationship (positive and negative) between expression of MIXTA and other genes involved in cannabinoid synthesis with expression of cannabinoids across the full seven weeks of flower development (Figure 1C). This analysis shows that *CsMIXTA* has a significant positive correlation with the expression of the two genes (*Geranyl Pyrophosphate Synthase* (*GPPS*) and *Olivetolic Acid Cyclase* (*OAC*)) required to generate the substrates (GPP and OA, respectively) necessary for the synthesis of the CBG, the first cannabinoid in the biosynthetic pathway. This analysis indicated that during flower development, *CsMIXTA* expression was also positively correlated with cannabinoid levels and expression of *Cannabichromenic Acid Synthase* (*CBCAS*) which generates the end product cannabinoid CBCa (Figure 1C). This analysis indicates that through coordinating trichome development, *CsMIXTA* may play an important role in cannabinoid biosynthesis.

**Figure 2:**
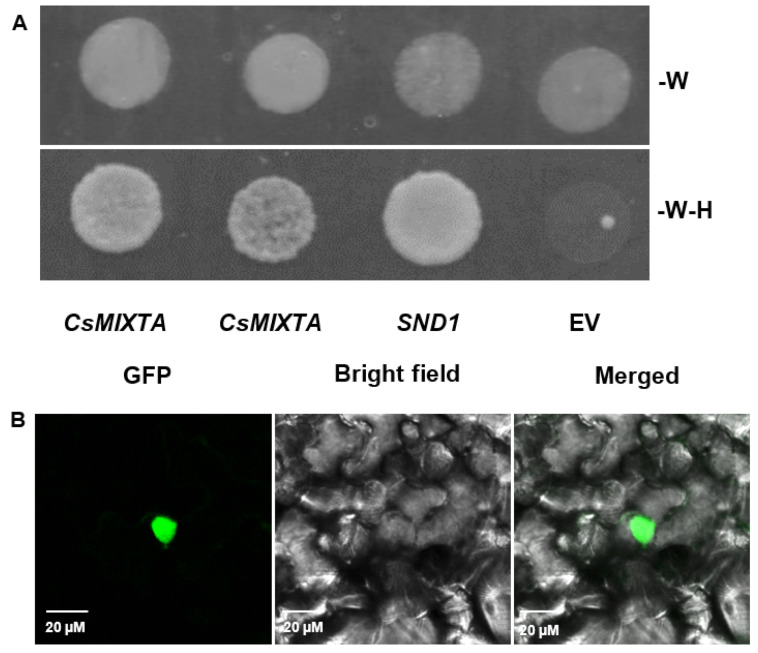
Yeast transactivation assay and subcellular localization of CsMIXTA. (**A**) Top: yeast cells grown on SD medium lacking W. All cells grew normally, including cells transformed with the empty vector (EV). Bottom: yeast cells grown on SD medium lacking tryptophan (W) and histidine (H). Cells expressing CsMIXTA produced healthy cultures, while pAS2 empty vector transformed culture did not. (**B**) *N. benthamiana* epidermal cells expressing a CsMIXTA-YFP fusion protein, observed using Nikon A1R Confocal microscope with excitation at 488 nm. YFP signal clearly indicates nuclear localization.

*CsMIXTA* belongs to the R2R3-MYB TF family and showed homology with the known R2R3-MYB and MIXTA-like TFs from other plant species. Using a yeast activation assay, we demonstrated that *CsMIXTA* is capable of transcriptional activation (Figure 2A). Transient expression of *CsMIXTA* in *N. benthamiana* leaves showed nuclear localization (Figure 2B). In some cases, we also observed plasma membrane localization of *CsMIXTA* (Figure S2).

### 2.2. CsMIXTA Promotes Glandular Trichome Formation in Tobacco Leaves

Our work monitoring *CsMIXTA* expression in cannabis shows evidence of an associative relationship between *CsMIXTA* expression during the female flower development and cannabinoid synthesis, presumably mediated by *CsMIXTA-*mediated control of trichome development. We undertook further work to examine if there is a causal relationship between *CsMIXTA* expression and trichome development in a heterologous system. Cannabis is recalcitrant to genetic transformation; a few reports have been published recently [16,17,18], but their transformation efficiencies are not robust. In lieu of a reliable cannabis transformation protocol, we used a heterologous system to determine if there is a causal effect of *CsMIXTA* expression on trichome morphogenesis. Tobacco (*Nicotiana tabacum)*, like cannabis, has glandular stalked trichomes but in the case of tobacco, GSTs are abundant on the leaves [19]. We ectopically expressed *CsMIXTA* (controlled by a constitutive (35S CaMV) promoter) in tobacco plants to evaluate if this cannabis TF could regulate glandular trichome initiation/development on tobacco leaves.

**Figure 3:**
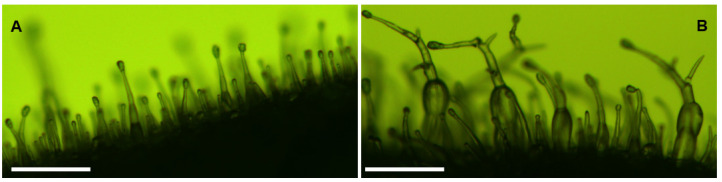
Trichomes on wt (**A**) and transgenic (**B**) tobacco leaf edges visualized using a dissection microscope. Samples were viewed at 38× magnification. It is noticeable that the base of some trichomes has become enlarged and trichomes are exhibiting novel branching in the transgenic line (**B**). Scale bars: 200 µm.

Figure 3 displays a side view of leaves from wt and a transgenic tobacco line, L1. An increase in trichome size and branching is clearly observed on the transgenic tobacco leaf. In addition, the upper leaf surface of the transgenic tobacco L1 is noticeably “hairier” than that of the wt, indicating a greater density of leaf trichomes produced in the *CsMIXTA* overexpressing tobacco line (Figure S3).

**Figure 4:**
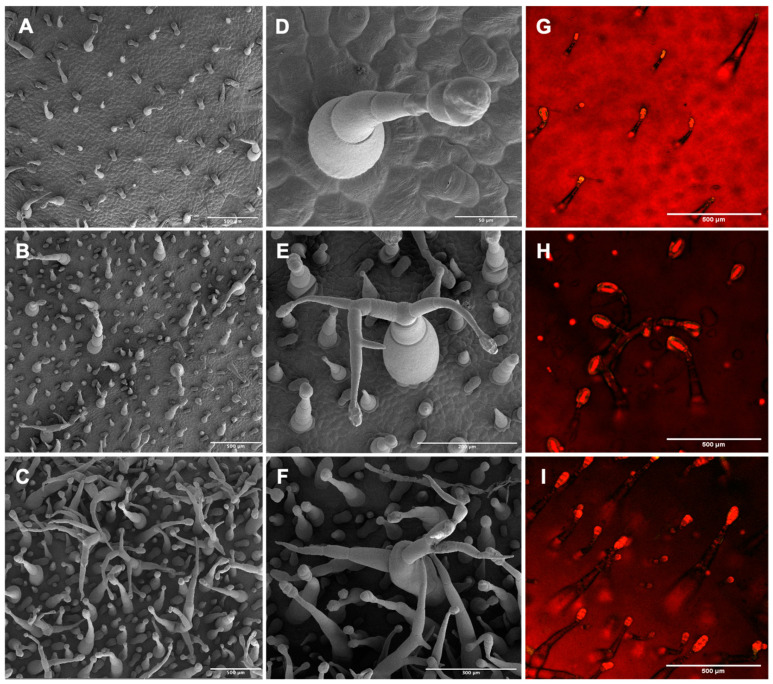
Scanning Electron Microscopy and Fluorescence Microscopy micrographs of wt and transgenic tobacco leaves. (**A**–**C**), SEM images of adaxial leaf surface of wt (**A**) and two transgenic lines L3 (**B**) and L1 (**C**). (**D**–**F**) Higher resolution micrographs of representative glandular trichomes from wt (**D**) and two transgenic lines L3 (**E**) and L1 (**F**). (**G**–**I**) Fluorescence Microscopy images of wt (**G**) and two transgenic lines L3 (**H**) and L1 (**I**) captured with a Nikon A1R confocal microscope. All samples were taken from tissue adjacent to the midrib of the third leaf from the apical meristem, and 2 cm from the petiole. In all cases, the images shown for each genotype are representative of other biological replicates. Fluorescence signals indicating glandular trichomes were counted from each tissue sample (*n* = 3). Scale bars in (**A**–**C**): 500 µm; in (**D**–**F**): 200 µm; in (**G**–**H**): 500 µm.

We further examined trichome morphology on tobacco leaves using SEM. Two transgenic lines (L1 and L3) were evaluated in this experiment (Figure 4). Both lines (Figure 4B,C) showed substantially higher trichome density compared to wt (Figure 4A). Typical wt trichomes possess only one gland and rarely branch [19]. However, we observed highly branched glandular stalked trichomes in both transgenic lines. In addition, trichomes in L1 showed higher density, size, and branching than L3, which could be due to the higher *CsMIXTA* expression in L1 (Figure S4). Close-up micrographs of representative highly branched glandular trichomes on the transgenic tobacco leaves (Figure 4E,F) and the regular wt glandular trichome are shown (Figure 4D). Floral organs of the transgenic tobacco plants were also investigated. Glandular trichomes are also formed on the abaxial side of the petal lobe (Figure S5). Anecdotal results showed that there were slightly more trichomes formed on the abaxial side of the petal lobe in the transgenic lines than those in wt, but no obvious structural difference was observed compared to what was seen in leaf glandular trichomes (Figure 3 and Figure 4).

**Figure 5:**
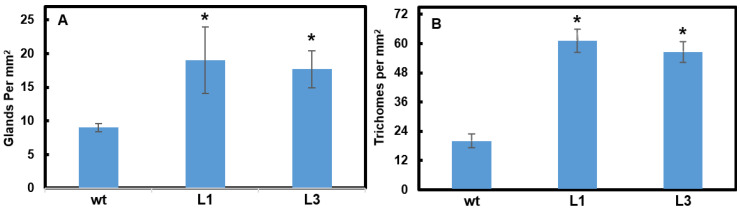
Tobacco transgenic lines overexpressing *CsMIXTA* generated more trichomes than wt. (**A**) Average number of glands on the glandular trichomes per mm^2^, observed by intrinsic fluorescence as shown in Figure 4G–I. (**B**) Average trichome number per mm^2^, obtained from SEM images in Figure 4A–C. Averages taken from 3 biological reps (*n* = 3) for each transgenic line and wt. * indicates *p* < 0.05, which was determined using Student t-test by comparing the two transgenic lines to wt.

Fluorescence Microscopy was used to produce intrinsic fluorescence images of the trichomes at the leaf surface (Figure 4G–I). Glandular trichome heads filled with secondary metabolites show strong fluorescence: multiple studies have used this method to quantify trichome gland number [10,12,13]. It is clear that the trichome heads were much larger in the two transgenic lines than those in the wt. Trichome counting based on the images obtained from Fluorescence Microscopy and SEM further showed that trichome glands (those that emitted yellow fluorescence in Figure 4G–I) in transgenic tobacco significantly increased nearly twofold compared to wt (Figure 5A); the total trichomes on the leaves of the transgenic tobacco lines significantly increased threefold compared to the wt (Figure 5B). The results indicate that *CsMIXTA* plays an essential role in glandular trichome initiation and development. Our findings provide evidence that *CsMIXTA* also functions in CSG cells to modulate trichome growth and development, consistent with the higher *CsMIXTA* expression detected in isolated female flower glandular trichomes (Figure 1A).

## 3. Discussion

In this study we identified *CsMIXTA*, the first characterized cannabis TF that could potentially influence cannabis CSG development. *CsMIXTA* is highly expressed in the female cannabis flowers, with expression patterns that are coordinated with cannabinoid biosynthesis and the development of inflorescences. Phylogenetic analysis indicated that *CsMIXTA* is closely related to *Arabidopsis thaliana* AtMYB16 and *Artemisia annua AaMIXTA* (Figure S1). MIXTA-like proteins have been shown to coordinate cuticle deposition in tomato and *Arabidopsis thaliana* [20,21]. Modification of *AaMIXTA* expression altered the biosynthesis of wax and cutin monomers, components of the cuticle [12], and MIXTA-like proteins identified in liverworts have implicated MIXTA in the early evolution of cuticle formation and therefore the colonization of land by plants [22]. Because cutin is an important structural component of the trichome, *CsMIXTA* may coordinate the biosynthesis of this compound in the trichome.

Our previous study demonstrated that expression of cannabinoid biosynthetic genes peaked in weeks 4 or 5 [15]; *CsMIXTA* expression showed a similar pattern. It has been shown that trichomes produce most cannabinoids during the latter capitate-stalked glandular phase. Therefore, it is reasonable to propose that the development of the stalk cells also occurs around week 4 of floral development, in correlation with the expression of *CsMIXTA*.

There is a strong positive correlation between *CsMIXTA* expression and the expression of both *GPPS* and *OAC*, enzymes which synthesize the necessary precursors (GPP and olivetolic acid, respectively) [15] for both cannabinoids and, in the case of *GPPS,* monoterpenes. Sessile (non-stalked) glandular trichomes produce minimal monoterpenes or cannabinoids before maturation, possessing a sesquiterpene-dominant chemotype. However, after the sessile trichomes develop into CSGs, cannabinoid and monoterpene biosynthesis takes place. Previous studies have shown that *GPPS* was more highly expressed in stalked trichomes, but *OAC* expression was similar among different glandular trichome types [10].

The heterologous overexpression of *CsMIXTA* in tobacco has shown a significant impact on trichome development with increased density, enlarged size and more branching (Figure 4). The overexpression of *CsMIXTA* in tobacco showed more distinct trichome phenotypes than that of *AmMIXTA* overexpression [14,23]. The overexpression of known MIXTA or MIXTA-like genes did not show a similar glandular trichome phenotype caused by overexpression of *CsMIXTA*, indicating that *CsMIXTA* may have unique functions in glandular trichome morphogenesis in cannabis. Figure 5 quantifies a modest (twofold) increase in trichome glands which can be seen in the Fluorescence Microscopy images (Figure 4G–I). What cannot be seen in the fluorescence images, however, is the forest of trichomes and trichome branches, which becomes evident in the SEM photos (Figure 4A–C). The increase in trichome density (threefold) as well as trichome branching indicates that *CsMIXTA* may also function to regulate the initiation and development of trichome types other than glandular stalked trichomes. According to these findings, genetic engineering of *CsMIXTA* may have the potential to promote cannabinoid production by increasing glandular trichome formation.

Our data provide evidence associating *CsMIXTA* with trichome development in cannabis. However, a definitive *causal* relationship between *CsMIXTA* expression, glandular trichome development, and cannabinoid production in cannabis is, at this point, beyond experimental verification due to the recalcitrance of cannabis concerning stable transformation. We also note that our work does not delineate the specific molecular steps in a *CsMIXTA* signaling cascade in cannabis. Future research is needed that might focus on the cell-to-cell signaling pathways by which glandular trichome development is generally coordinated, and on proteins and promoter elements which interact with *CsMIXTA* in *C. sativa.*

## 4. Materials and Methods

### 4.1. Cannabis Plant Growth

A hemp variety, Cherry Wine, was used in this study. Plants were acquired using cuttings from mother plants in our research greenhouses. Cuttings were treated with Hormodin powder and stuck in rockwool cubes soaked with 20 mL/L Clonex Nutrient Solution (Growth Technologies Ltd., Taunton, UK). Cuttings were allowed to root for 3 weeks before transplant. All plants were grown in #600 standard nursery pots filled with Promix-BX25 (Premier Tech Horticulture, Quakertown, PA, USA) soilless medium and Osmocote 15-9-12 (Scott’s Miracle-Gro, Marysville, OH, USA). Plants were grown under 16 h light/8 h dark conditions with supplemental lighting from high-pressure sodium lighting. The light intensity from the high-pressure sodium lights is 568 µmol m^–2^ s^–1^ nm^−1^. Photoperiod was changed to 12 h light/12 h dark using blackout curtains. Jack’s nutrient solution fed via drip fertigation.

### 4.2. Isolation of RNA and cDNA Synthesis

Different tissue samples were collected in the eighth week of flowering. Amounts totaling 100 mg of plant tissues were collected and immediately frozen in liquid nitrogen. The NucleoSpin Plant and Fungi RNA Isolation Kit (Macherey-Nagel) was used for RNA isolation according to manufacturer’s manual. cDNA was synthesized from 1 µg RNA using the iScript Reverse Transcriptase Master Mix (BioRad).

### 4.3. Isolation of Cannabis Trichomes

Trichome isolation was performed following the protocol developed by Livingston et al. (2020) [10] with slight modifications. Isolation buffer was made as described by Livingston et al. but excluding Amberlite. Approximately 3 g of fresh inflorescence tissue was used for trichome isolation. After isolation, the tube containing approximately 100–300 mg of trichome tissue enriched with glands was then frozen at −80 °C until RNA isolation.

### 4.4. Analysis of CsMIXTA Expression Using qPCR

qPCR analysis was performed using Bio-Rad CFX. iTaq Universal Sybr Green Master Mix was used (Bio-Rad). For all qPCR reactions, *CsUbiquitin* was used as the internal reference [24]. The *CsMIXTA* Primers were forward 5′-TCCATGCTTTACTAGGCAACAG-3′, reverse 5′-CCACCGTCTTGTTGAGAGAG-3′. Experiments were performed with four biological replicates.

### 4.5. Molecular Cloning of CsMIXTA

*CsMIXTA* was cloned out of cDNA using iProof HF Master Mix (Bio-Rad). The primers for cloning were forward 5′-CAGTCGACTGGATCCGGTACCATGGGTCGGTCACCATGCTG-3′, reverse 5′-GAAAGCTGGGTCTAGATATCTCGAGAACATAGGAGAATCTG-3′. NEBuilder HiFi DNA Assembly kit (New England Biolabs) was used for the cloning of *CsMIXTA* into KpnI and XhoI digested pENTR-3C vector (Invitrogen, Waltham, MA, USA). LR reaction was then performed to clone *CsMIXTA* into the pB7YWG2 binary vector with a 35S CaMV promoter using LR Clonase II (Invitrogen), as well as the pAS2 and pUBC vectors.

### 4.6. Agrobacterium Mediated Transformation of Nicotiana tabacum

*N. tabacum* (tobacco) seeds were acquired from Dr. Yi Li at the University of Connecticut. Seeds were sterilized using 3% bleach and 70% ethanol and planted in sterile magenta boxes containing 50 mL full strength Murashige and Skoog (MS) salts (Caisson Labs, Smithfield, UT, USA), MES (adjusted to pH 5.7 with Tris), 1% sucrose, and 1% agar. Seedlings were grown under fluorescent lighting in a growth chamber at 28 °C with a 16-h photoperiod. Leaves from 3-week-old sterile tobacco plants were used for transformation by *Agrobacterium tumefacience* GV3101 harboring pB7YWG2-*CsMIXTA*. Tobacco leaf disc transformation was performed as described previously (Zheng et al., 2007). Rooted transformants were transferred to Promix-BX amended with 12 g/gal Osmocote 15-9-12 and maintained in the greenhouse with 16 h photoperiod.

### 4.7. Yeast Transcriptional Activation Assay

*CsMIXTA* was fused with the GAL4-BD domain in the pAS2 vector using LR Clonase II (Invitrogen). The resulting plasmid was transformed into yeast strain AH109 (Clontech, Mountain View, CA, USA) using the Frozen-EZ Yeast Transformation II Kit (Zymo Research, Irvine, CA, USA). A known Arabidopsis TF, SECONDARY WALL-ASSOCIATED NAC DOMAIN PROTEIN 1 (*SND1*) (provided by Dr. Huanzhong Wang), was used as a positive control and empty vector as a negative control. The transformed cells were plated on synthetic defined (SD) media to select positive transformants. A 4 μL amount of yeast culture harboring corresponding constructs was dropped onto a solid SD medium with or without histidine to check for activation of reporter genes. Yeast growth was observed and documented after cultivation at 28 °C for 2 days.

### 4.8. Transient Expression of CsMIXTA in N. benthamiana Leaves

*CsMIXTA* was fused to YFP using a ubiquitin promoter-containing plasmid (pUBC-YFP) and LR Clonase II (Invitrogen), and the resulting construct was transformed into *Agrobacterium tumefacience* GV3101. Transient transfection protocol was performed as described by Espinoza-Patharkar et al., modified from [25].

### 4.9. Scanning Electron Microscopy

Squares of tobacco leaf tissue 3 mm in size were excised with midribs and the veins were removed. These squares were fixed for 24 h in formaldehyde-acetic acid-ethanol (10% formaldehyde, 5% acetic acid, 50% ethanol, 35% MilliQ H_2_O). These samples were then transferred to a 70% ethanol solution twice, and then dehydrated through a graded ethanol series. Samples were collected and placed into stainless steel containers for critical point drying while completely submerged in 100% ethanol. Dehydrated and dried samples were mounted onto SEM stubs using double-sided carbon tape, and sputter coated with gold nanoparticles. The samples were mounted into a Nova Nanosem 450 and imaged.

### 4.10. Fluorescence and Confocal Microscopy

Samples were mounted between two cover slips and viewed using a Nikon A1R confocal microscope through a 10× Plan Apo lens. Both channels were excited at 488 nm. Emissions were collected with a GFP filter (488 nm) as well as a red filter (700 nm). Z-stacks were collected at a step size of 27 microns. Composite channel/stack images/scale bars were produced in ImageJ. Trichomes were counted in photographs using “cell counter” in ImageJ.

## 5. Conclusions

Overexpression of the cannabis TF *CsMIXTA* in tobacco resulted in an increase in trichome density, size, and branching on the leaves of multiple transgenic lines, demonstrating that *CsMIXTA* is involved in trichome initiation and development. In cannabis, *CsMIXTA* is strongly expressed in inflorescence tissue and has an expression pattern which coordinates with key cannabinoid biosynthesis genes during female flower development. Through analyzing cannabis gene expression data, we established an association between the expression of *CsMIXTA* and cannabinoid biosynthetic genes as well as flower/trichome development. *CsMIXTA* is also highly expressed in the isolated trichome, suggesting a function within the trichome. *CsMIXTA* expression has a significantly positive correlation with the expression of *GPPS* and *OAC*: two key enzymes in the cannabinoid biosynthesis pathway. These data suggest that *CsMIXTA* not only regulates glandular trichome morphogenesis, but also cannabinoid and/or cutin biosynthesis. Together, these data create a strong case to support the hypothesis that *CsMIXTA* is a TF involved in the coordination of CSG development in female cannabis flowers; increasing *CsMIXTA* expression in cannabis could potentially enhance cannabinoid production in female flowers.

## 6. Patents

A provisional patent application has been submitted based on the findings in this report.

## Data Availability

Not applicable.

## Supplementary Materials

The following supporting information can be downloaded at: https://www.mdpi.com/article/10.3390/plants11111519/s1, Figure S1: Phylogenetic tree of MIXTA and MIXTA-like genes; Figure S2: Confocal microscopy showing *CsMIXTA* localization; Figure S3: photographic comparison of upper leaf surface in WT and transgenic tobacco (line 1); Figure S4: qPCR results showing CsMIXTA expression in Transgenic lines 1, 3, and 6 vs. WT; Figure S5: SEM micrographs of trichomes on the abaxial side of the petal lobe in WT and transgenic lines 1 and 3.

## Accession Numbers

The accession numbers for proteins in the phylogenetic tree are: CsMIXTA, XP_030485058.1; CsMIXTA2, XP_030481714.1; AaMIXTA1, ALD84252.1; AtMYB16, NP_197035.1; AtMYB106, NP_186763.2; AtMYB17, OAP04938.1; NtMYB39, XP_016468968.1; AmMIXTA1, CAA55725.1; AmMYBML1, CAB43399.1; GhMYB25, AAK19616.1.
