# Supplementary material for: Overexpression of CsMIXTA, a Transcription Factor from Cannabis sativa, Increases Glandular Trichome Density in Tobacco Leaves
Source: Plants (Basel). 2022 Jun 6;11(11):1519. doi: 10.3390/plants11111519 (PMC9182785; doi:10.3390/plants11111519)
Supplement: Supplementary file 1 [file plants-11-01519-s001.zip › plants-1736329-supplementary.pdf]

plants

Supplementary Material

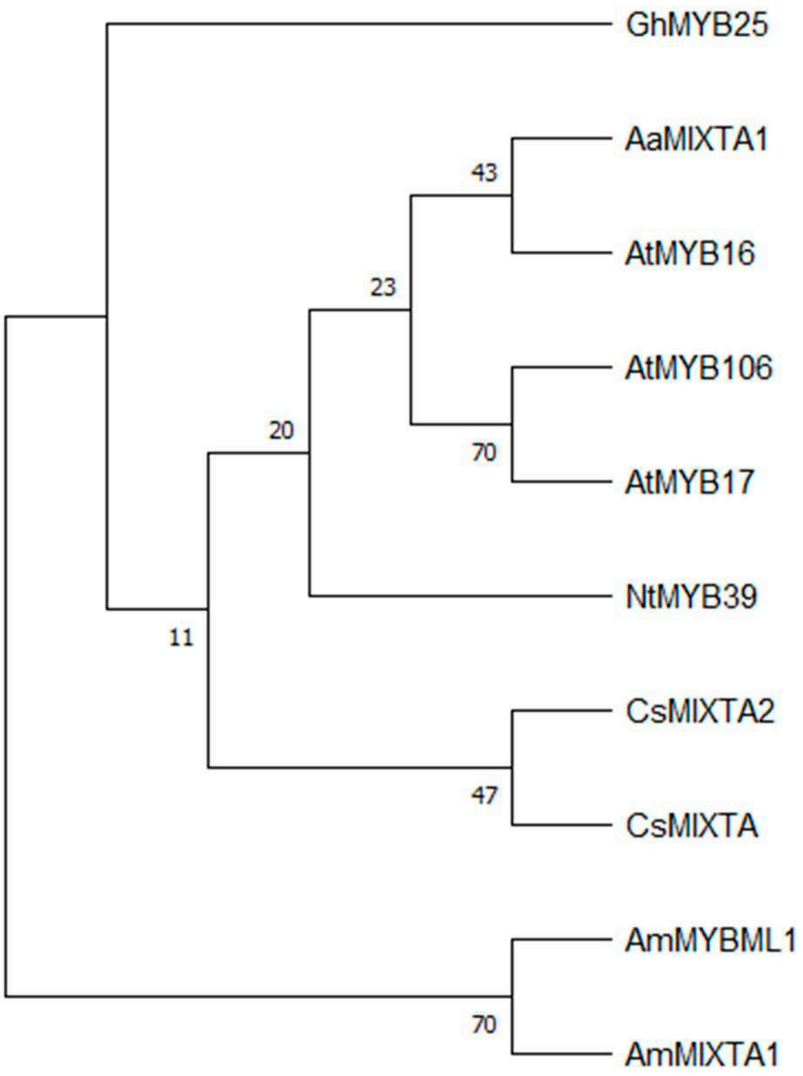

Figure S1. Phylogenetic tree of MIXTA and MIXTA-like genes. CsMIXTA1 is the gene studied in this report. CsMIXTA2 is the homolog of CsMIXTA1. CsMIXTA1 and CsMIXTA2 are closely related to AtMYB16 and AaMIXTA1, suggesting their functional similarity. The tree was generated using MEGA X.

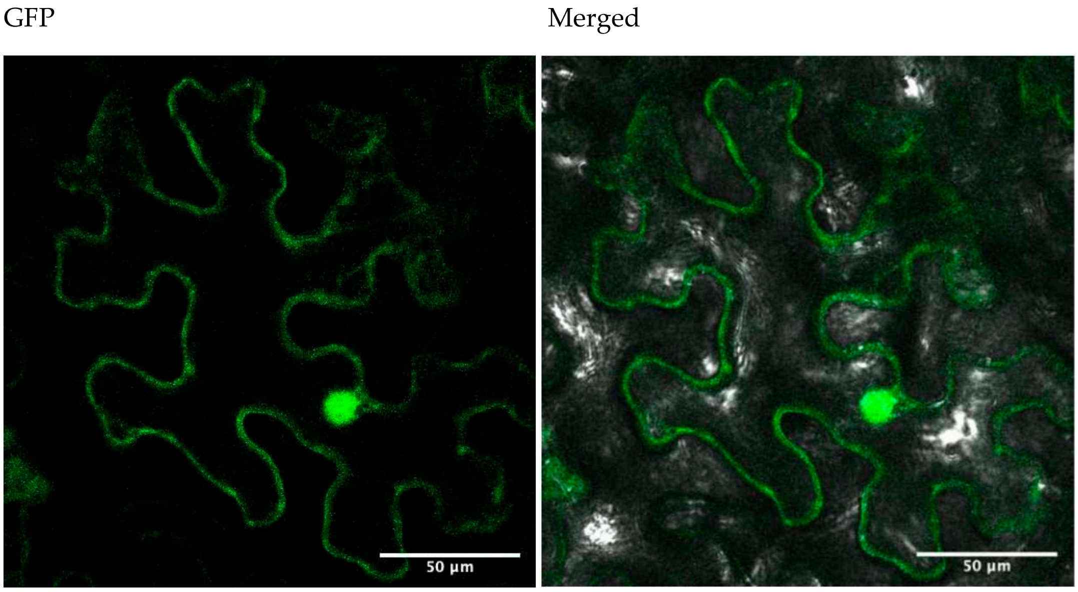

Figure S2. Confocal microscopy showed that CsMIXTA can be localized both on the plasma membrane and in the nucleus.

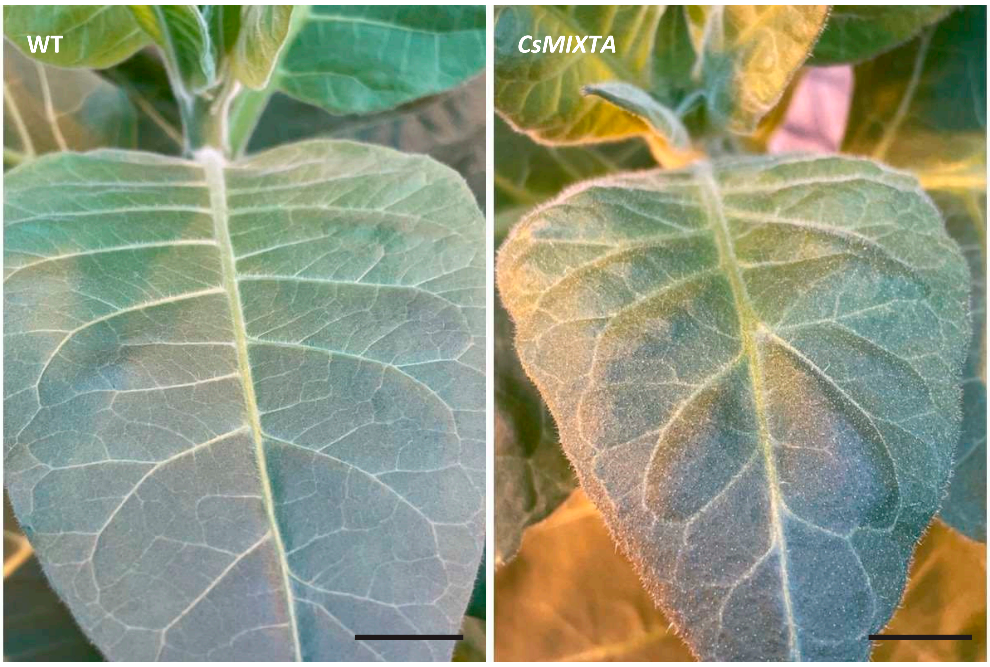

Figure S3. Comparison of upper leaf surface in wt (left) and transgenic tobacco line1 expressing *CsMIXTA* (right). It is noticeable that *CsMIXTA* overexpressing line showed a hairier surface due to the higher density of trichomes produced. Scale bars represent 50 mm.

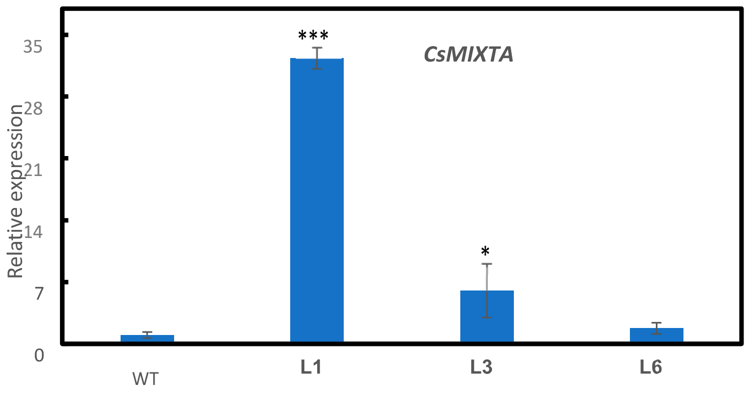

Figure S4. qPCR results showed *CsMIXTA* expression in transgenic lines L1, L3, and L6 compared to wt. L1 and L3 showed significantly higher CsMIXTA expression. * indicates P < 0.05 and *** indicates P < 0.001, which was determined using Student t-test.

WT L1 L3

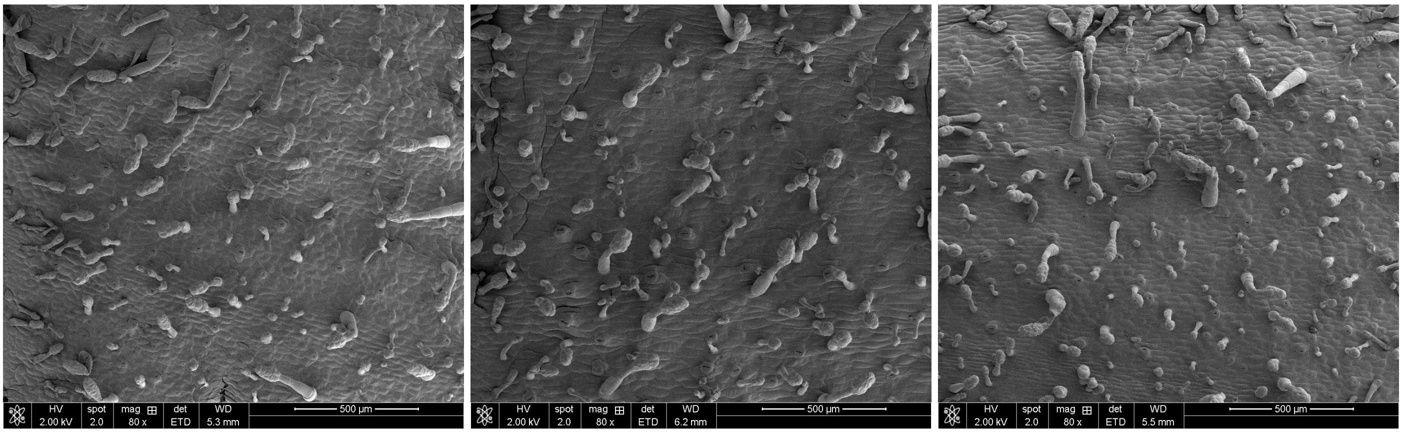

Figure S5. SEM micrographs of trichomes on the abaxial side of the petal lobe in wt, transgenic tobacco L1 and L3. There are slightly more trichomes produced in the transgenic lines than in the wt. It is noticeable that most the trichomes on the abaxial side of the petal lobe are glandular trichomes.
